# Building While Responding: Moldova’S Experience in Developing Clinical Surge Capacity for Radiation Emergency Response

**DOI:** 10.1017/dmp.2025.72

**Published:** 2025-04-15

**Authors:** Stetsyk Vitalii, Ion Apostol, Nera Belamarić, Cornelia Panico, Grbic Miljana, Kenbaeva Zhanat, Tereshchenko Hanna, Anatolii A. Chumak

**Affiliations:** WHO Regional Office for Europe; National Agency for Public Health, Ministry of Health, Republic of Moldova; Croatia; WHO Country Office in Republic of Moldova; Republic of Moldova; Department of Environment, Climate Change and Health, Division of Healthier Populations, World Health Organisation - Geneva, Switzerland; WHO Country Office in Republic of Moldova; National Research Centre for Radiation Medicine, Hematology and Oncology of the National Academy of Medical Sciences of Ukraine

## Abstract

To enhance radiological and nuclear emergency preparedness of hospitals while responding to the refugee crisis, the Government of the Republic of Moldova implemented an innovative approach supported by the World Health Organization (WHO). This initiative featured a comprehensive package that integrated health system assessment, analysis of existing plans and procedures, and novel medical training component. The training, based on relevant WHO and International Atomic Energy Agency (IAEA) guidance, combined theory with contemporary adult learning solutions, such as practical skill stations, case reviews, and clinical simulation exercises.

This method allowed participants to identify and address gaps in their emergency response capacities, enhancing their ability to ensure medical management of radiological and nuclear events. This course is both innovative and adaptable, offering a potential model for other countries seeking to strengthen radiological and nuclear emergency response capabilities of the acute care clinical providers.

In 2024, nearly 300 million people were affected by emergencies worldwide, a staggering fourfold increase compared to 2014.^1^ The WHO has raised alarms about the escalating humanitarian health needs, which are compounded by factors such as worsening security, sociodemographic vulnerabilities, climate change, and the protracted impacts of the COVID-19 pandemic on global health systems.^2^ Moldova is not exempt from these harsh realities. The country, still grappling with the aftereffects of the pandemic, has faced unprecedented challenges, including the influx of refugees from Ukraine resulting from the ongoing military conflict with the Russian Federation.

The refugee crisis began in February 2022, caused by the Russian invasion of Ukraine, one of the largest conflicts in Europe in recent history. By March 2024, over 6.4 million Ukrainians were recorded as refugees globally, with Moldova being among the primary hosts despite its fragile economy and its status as one of Europe’s most disadvantaged nations.^3^ According to the United Nations High Commissioner for Refugees (UNHCR), nearly one million refugees have arrived in Moldova from Ukraine, making it one of the most heavily affected countries in Europe.^4^ The number of refugees represents 39% of Moldova’s population of 2.53 million, highlighting the immense strain on the country’s resources.^5^

In response to this crisis, Moldova has received considerable support from international organizations and United Nations (UN) entities, which have assisted the Government in strengthening its emergency preparedness and response capacities. Among the priority hazards identified in Moldova’s national emergency response plan is the risk of radiological and nuclear (RN) emergencies, particularly given the proximity of the South Ukraine Nuclear Power Plant, located just 100 km from Moldova’s border. The unpredictable Russian airstrikes on Ukraine’s energy infrastructure have spurred the Moldovan government to take comprehensive preparedness actions, starting as early as 2022.^6^

This paper captures the collaborative efforts of the Moldovan government, WHO, and other national and international stakeholders in enhancing the readiness of hospitals through targeted capacity-building initiatives for healthcare providers and the establishment of enabling systems, including policies, plans, procedures, and supplies. These interventions are interlinked with broader reform of the emergency care system in Moldova.

While numerous training packages, guidelines, and tools are available online for policymakers working to strengthen health systems for specific emergency responses, there is limited documented experience in applying WHO and IAEA trainings, tools, and guidelines to real-world crises. Furthermore, there is a limited availability of training for medical workers on particularities of RN response. This paper demonstrates how various tools and guidelines can be effectively utilized throughout the emergency preparedness cycle to rapidly enhance a country’s readiness for RN hazards. Moldova’s experience offers valuable lessons for other nations facing similar threats, especially in the context of an increasingly unstable global security environment.

## Brief introduction to the context

Globally, nuclear events are rare, but their consequences can be devastating.^7^ The medical response to radiation emergencies is highly specialized, and the psychological pressure on both the public and medical personnel can significantly impact the implementation of protective measures and the overall effectiveness of the medical response, sometimes leading to substantial “non-radiological effects.”^8^ Adequate training of medical personnel is crucial to ensure a timely and effective response, which in turn promotes appropriate behavior among patients and the public.

The emergency preparedness and response (EPR) planning system in Moldova is structured on a multilevel and all-hazard approach, as outlined in the Law on Civil Protection.^9^ This approach ensures that contingency plans are developed based on risk assessments. The WHO, through its regional and country offices, has been a crucial partner in supporting the Moldovan health sector during various crises, including the COVID-19 pandemic and the ongoing Ukrainian refugee crisis.

In light of new challenges and emerging threats due to the ongoing conflict in Ukraine, Moldova recently reviewed its EPR arrangements. The civil protection system in Moldova has identified three key types of radiation hazards in Ukraine that could have direct or indirect impacts on Moldova: ^10^

- Nuclear emergencies in a neighboring country – involving the release of radioactive materials into the environment, such as accidents or attacks at nuclear power plants with a consequent influx of refugees or evacuees who could be potentially contaminated with radioactive substances;
- Detonation of contaminated weapons (e.g., dirty bombs) or other malicious acts, with a consequent influx of refugees or evacuees who could be potentially contaminated with radioactive substances;
- Radiological emergencies related to inappropriate use, storage, or transportation of radioactive sources, or their deliberate use.

Historically, Moldova has not prioritized these scenarios in its national emergency planning, as the country does not have large nuclear programs. Consequently, the need has been recognized to develop contingency plans, training programmes, and acquire radiation equipment to adequately detect and respond to radiation emergencies. The most critical challenge identified in this context is the preparedness of the medical sector at the hospital level.

To address this problem, Moldova’s medical sector must be trained and equipped to handle mass casualties resulting from radiation exposure, starting with prehospital care at border crossings and extending to specialized medical care at designated hospitals. This includes developing specific protocols, acquiring the necessary equipment, and providing specialized training to healthcare professionals to ensure a robust response to potential radiation emergencies.

In response to these challenges, the Moldovan Ministry of Health (MoH) has sought methodological and technical support from the WHO, the European Commission (EC)’s Health Security Committee, and other experienced development partners. Over the past two years, under the leadership of the WHO Country Office in the Republic of Moldova (WCO), significant resources have been mobilized from the Governments of the United States of America, Norway, Switzerland and the European Union. Subsequently, the National Agency for Public Health (NAPH) and the Center of Prehospital Emergency Medicine, have been equipped with a Mobile Radiological Lab, personal protective equipment (PPE), radiation detectors, and other essential response materials. Additionally, substantial support has been provided to the prehospital level through the European Network of CBRN (Chemical, Biological, Radiological, and Nuclear) Centers of Excellence and Swiss Cooperation Office in Moldova. This support has included the acquisition of radiological equipment and PPE, followed by extensive personnel training. Between June 1, 2023, and July 31, 2024, five “Train-the-Trainer” sessions and exercises were conducted for 198 trainees at the prehospital level.^11^ Specialized medical response to radiation events at the hospital level, particularly for personnel from MoH-designated hospitals, also required pertinent training. The first of these training sessions was conducted in March 2024 with the support of the WHO Country Office, where a group of international experts shared their knowledge with 41 doctors from CBRN-designated hospitals.

In addition to capacity building, Moldova is making significant progress in establishing national stockpiles of medical countermeasures for radiation emergencies. The NAPH now has the capacity to equip 3,000 first responders from various national response agencies with personal dosimeters, ensuring accurate monitoring and data management of their exposure to ionizing radiation during emergency activities.^12^ Additionally, a stockpile of stable iodine tablets has been established at NAPH subdivisions, ready for distribution as needed.^13^ However, the development of stockpiles for specialized medicines required for radionuclide decorporation and the treatment of patients overexposed to radiation is still ongoing.

To ensure a cohesive and comprehensive approach, the WHO Country Office in Moldova continues to support the MoH in drafting a contingency plan for the national health sector’s response to RN emergencies. This plan will integrate all necessary components to enable an efficient and effective response.

## Discussion

Achieving readiness for RN hazards requires a comprehensive, system-wide approach involving multiple disciplines. The implementation of various scientific tools and frameworks is essential to ensure that preparedness measures are robust and effective.

Several tools are used to assess and enhance the readiness for RN hazards. Among these, the WHO’s Joint External Evaluation (JEE) and the State Party Self-Assessment Annual Reporting (SPAR) tools are critical for reviewing a country’s capacity to handle such emergencies.

1. The JEE provides a structured process for assessing a country’s ability to prevent, detect, and respond to public health threats, including RN hazards. JEE’s section on Radiation Emergencies highlighted the need to work on improving the detection capabilities of public health responders, to develop further contingency planning and national regulations and responder-level operational procedures, and to introduce evidence-based training for public health and healthcare workforce on RN response.^14^
2. As mandated by Article 54 of the International Health Regulations (IHR, 2005)^15^, and the World Health Assembly (WHA) resolution 61.2,^16^ Moldova submits an annual SPAR, which outlines the progress in achieving core public health capacities, including those related to radiation and chemical safety.^17^ The SPAR tool involves grading the maturity level of various capacities by each state party. For the radiation emergency preparedness indicator (C15), the country has assessed itself at level 3 out of 5. This level indicates access to specialized healthcare for radiation injuries and laboratory testing capacity for radiation monitoring. However, the report also identifies the ongoing need for regular formal exercises, reviews, and evaluations of radiation emergency arrangements, as well as improved coordination between national radiation authorities, health, and non-health sectors at all stages of preparedness and response.
3. Strategic Toolkit for Assessing Risks (STAR) was applied in June 2024.^18^ This assessment involved 50 participants from 20 government ministries and specialized agencies, who evaluated 32 hazards and identified key preparedness actions. The STAR assessment highlighted nuclear and chemical hazards as high-risk areas, underscoring the need for targeted preparedness measures to be constituted.

## Development of innovative simulation-based training for clinicians on rn emergency response

A key component of Moldova’s preparedness strategy is the development of innovative, simulation-based training programs for clinicians. These educational programs are designed to enhance the readiness of medical professionals to respond effectively to nuclear or radiological emergencies. The training curricula aim to strengthen both prehospital and hospital readiness, ensuring that medical doctors, nurses and other healthcare professionals are equipped with the necessary knowledge and skills to manage radiation-related emergencies. Given the limited availability of the courses specific to the context of Eastern Europe, WHO and MOH embarked on the development of a new course blending available international guidance and tools with adult simulation-based learning methods.

Most of the material for these workshops was developed specifically for this purpose, drawing from WHO, IAEA, and European Union (EU) documents, guidance, and training materials.^19^ Despite the critical importance of being prepared for RN emergencies, there has historically been a lack of comprehensive training packages for clinical workers within WHO’s educational materials, with a focus more on developing guidance and public health countermeasures than on delivering exhaustive training sessions.

However, WHO supports countries in strengthening their national capacities through its Collaborating Centres and global expert networks, such as the Radiation Emergency Medical Preparedness and Assistance Network (REMPAN) and BioDoseNet. While WHO provides technical guidance and tools for the examination of internal contamination, thyroid monitoring, establishing national stockpiles, and integrating mental health into emergency responses, the training aspect is often conducted on an ad-hoc basis, typically in response to requests from specific countries.^20^

Training in the medical response to RN emergencies has usually been the responsibility of national authorities or the IAEA. The IAEA collaborates with WHO in providing policy advice and technical support for national health authorities under the framework of the Inter-Agency Committee for Radiological and Nuclear Emergencies (IACRNE).^21^ Countries operating nuclear power plants and thus facing a greater possibility of a major emergency, often develop their own training programs. However, many countries with limited financial or human resources rely on international assistance to build their capacities.

Globally, the IAEA plays a central role in promoting safety in the application of nuclear energy and protecting human health and the environment against ionizing radiation. It offers a variety of training courses at national, regional, and international levels. European countries have also established associations and platforms, such as HERCA (Heads of the European Radiological Protection Competent Authorities) and the NERIS (Nuclear and Radiological Emergency Response and Recovery) Platform, which focuses on preparedness and response to RN emergencies. These platforms facilitate dialogue and methodological development among European organizations.^22,23^

In regions outside Europe, networks such as the Forum of Nuclear Regulatory Bodies in Africa, the Asian Nuclear Safety Network, the Arab Network of Nuclear Regulators, and the Ibero-American Forum of Radiological and Nuclear Regulatory Agencies also include thematic areas on emergency preparedness and response.^24,25,26^ However, none of these platforms specifically focus on the medical response to radiation emergencies or provide dedicated training materials on the topic for Eastern European non-EU states.

Moldova’s experience in developing and implementing simulation-based training for clinicians on RN emergency response is a significant step forward. It not only enhances the country’s preparedness but also contributes valuable insights that can be shared with other countries facing similar challenges.

## Novel approach to RN emergency preparedness in moldova

The workshop conducted in Moldova was distinctive for its comprehensive agenda and content, focusing on the clinical aspects of the response to RN emergencies. It covered all phases of emergency management—preparedness, response, recovery, and long-term follow-up for affected populations. The workshop, which spanned three days, was designed to be highly interactive and practical. It included theoretical lectures, group work, and a simulation exercise using the EMERGO Train System (ETS).^27^

It was conducted in Chisinau from March 5 to March 7, 2024, and gathered 33 participants from the National Agency for Public Health and medical professionals from the facilities dedicated to the provision of care to victims of RN emergencies. The teaching faculty consisted of experienced WHO, IAEA and national experts from Croatia, France, Moldova, Slovenia, Ukraine.

## Theoretical sessions and expert insights

The theoretical part of the training began with presentations on Moldova’s national response plans and capabilities for RN emergencies. Participants were introduced to the international framework for emergency preparedness and response, which included the roles of WHO and other international organizations, as well as the mechanisms for inter-agency coordination.

Subsequent lectures covered a range of topics, starting with the various uses of radiation sources, types of radiation emergencies, and radioprotection principles. These foundational topics were included to refresh the knowledge of medical professionals who do not regularly work with radiation sources and may therefore not receive the latest information on radiation protection programs.

More specialized presentations followed, addressing the biological and health effects of radiation, exposure assessment, dosimetry methods, contamination and decontamination procedures, trauma and radiological triage, and the clinical management of radiation injuries, such as Acute Radiation Syndrome (ARS) and Cutaneous Radiation Syndrome (CRS). The participants were also briefed on best practices for prehospital care, hospital preparedness, stockpile management, and the formation of checklists based on the existing clinical resources of their healthcare facilities. The mental health impact of radiation emergencies and risk communication were also important topics covered during the training.

The workshop included case studies from past RN emergencies, such as the Chornobyl and Fukushima nuclear accidents and the Goiânia radiological incident, which are among the most severe radiation emergencies in history. Prof. Anatolii Chumak, a notable expert who was part of the response team during the Chornobyl accident, provided a first-hand account of managing the immediate and long-term consequences of such extreme events. This account of direct experience with a critical emergency added significant value to the training, highlighting the challenges medical personnel may face in real-life scenarios.

## Interactive sessions and clinical pathway’s simulation exercise

The workshop’s interactive sessions (skill stations) were conducted in small groups, focusing on the development of practical skills, such as using PPE and radiation measuring devices, wound decontamination, trauma and radiological triage, and development of hospital-response plans to a nuclear power plant emergency scenario. These sessions provided hands-on experience and reinforced the theoretical knowledge gained during the lectures.

A key highlight of the workshop was the use of the EMERGO Train System (ETS), a tabletop simulation method that allows participants to engage in disaster simulation of clinical pathways through role-playing exercises on whiteboards, supported by patient cards. Participants managed a simulated mass casualty event, performing roles as they would in a real emergency—conducting triage, decontamination, managing emergency departments, and coordinating with other stakeholders. This simulation allowed participants to identify and address potential weaknesses in their contingency plans while dealing with simulated patients during the emergency.

The simulation exercise, which lasted for one and a half hours, was well received by participants. They were divided into prehospital and hospital response teams, with each further subdivided into specific task forces. The exercise was based on a predetermined scenario that tested the participant’s ability to respond effectively under the pressure of limited time and resources. The use of simulation provided a realistic training experience, enabling participants to better understand the complexities and limitations of managing a radiation emergency and gain lessons for further improvement of their response plans.

## Language and accessibility

To ensure that all participants could fully engage with the material, all presentations and interactive session materials were translated into Romanian in advance. The workshop was conducted in English, with simultaneous translation into Romanian, ensuring clear communication and understanding of all the content delivered.

## Comparison with international programmes

There are few, if any, analogs to this type of comprehensive and interactive training within the WHO’s existing programs. While some EU countries have conducted similar training initiatives, these have often lacked the simulation exercise component that was a central feature of the Moldovan workshop.

Moldova, through its participation in the EU CBRN Centers of Excellence network project, has recognized the critical need for training general hospitals and nominating at least four national reference hospitals with advanced capacities in specialized care for overexposed and internally contaminated individuals.

This workshop marked a significant step in building the national capacity in Moldova to respond to RN emergencies, setting a precedent for other Member States looking to enhance their preparedness in this critical area.

## Analysis of feedback

The workshop concluded with a quiz and a written evaluation to assess the effectiveness of the training.

The quiz conducted at the end of the workshop indicated a significant increase in participants’ knowledge of radiation emergency medical response, confirming that the core messages of the workshop had been effectively communicated.

The evaluation consisted of 18 questions, asking participants to rate their satisfaction with various aspects of the workshop, including the overall organization, the venue, workshop materials (such as handouts and slides), audiovisual equipment, and technical setups. Participants also assessed the quality of the lectures and presentations, the effectiveness of the interactive activities, group discussions, and the Emergo train system used during the simulation exercise. Additionally, they were asked to evaluate the level of engagement and interaction of the lecturers with the participants. Finally, the survey inquired whether the overall workshop experience and content aligned with participants’ expectations and needs.

Because some participants needed to leave before the workshop concluded, a total of 26 participants (out of 30 present on the last day) completed the evaluation. Among them, 92% expressed high levels of satisfaction. Based on these results and oral feedback provided during the workshop, it can be concluded that the workshop was well received and successfully managed.

## Future plans for strengthening RN emergency preparedness

Building on the recent advancements in Moldova’s EPR capabilities, the focus moving forward will be on further strengthening the health sector’s readiness for RN emergencies. Key aspects of the future plan include:

### 1 Development of Comprehensive Training Programs

The success of the recent workshop in Moldova highlights the need for ongoing education. In collaboration with the WCO and international partners, Moldova plans to develop dedicated training programs tailored to various levels of responders, including prehospital care providers, hospital staff, and national response agencies. These programs will encompass all aspects of medical response to RN emergencies, incorporating both theoretical knowledge and practical simulation exercises. This approach ensures that all levels of responders will be adequately prepared to handle such emergencies.

### 2 Regional collaboration and knowledge sharing

Moldova will explore opportunities to develop a regional training product based on the experience gained from the recent workshop. This initiative could involve creating a standardized training package that can be utilized by other countries in the region facing similar risks. Collaboration with WHO and IAEA will be essential in ensuring that the training material is current, relevant, and adaptable to different contexts.

### 3 Enhancement of national contingency plans

The Moldovan MoH will continue to work with WHO and other partners to finalize and implement the draft contingency plan for the health sector’s response to RN emergencies as well as mapping clinical pathways of patients with radiation induced injuries. This plan will be integrated into the broader national emergency response framework, ensuring a coordinated and effective response in the event of an incident. The aim is to create a comprehensive plan that addresses all stages of emergency management, from preparedness to recovery.

### 4 Strengthening national stockpiles

Efforts will be made to further develop and maintain national stockpiles of medical countermeasures for radiation emergencies. This includes expanding the inventory of personal dosimeters, stable iodine tablets, and specialized medicines required for the decorporation of radionuclides and treating patients exposed to radiation. Ensuring these supplies are readily available and properly managed is crucial for a swift and effective response.

### 5 Regular drills and exercises

To ensure that the health sector remains prepared for RN emergencies, Moldova will conduct regular drills and exercises, including joint exercises with neighboring countries and international partners. These activities will help identify gaps in preparedness and response and provide opportunities for continuous improvement. Regular practice is vital for maintaining readiness and ensuring that all involved parties can coordinate their responses effectively during an actual emergency.

### 6 JEE and continuous monitoring

Moldova will undertake periodic reviews of its emergency preparedness capabilities, including updating the JEE to reflect recent developments and emerging threats. Continuous monitoring and evaluation will be crucial to maintaining a high level of readiness. An updated JEE will provide a more accurate assessment of the country’s capabilities and help identify areas for improvement.

## Limitations

Despite the significant progress made, several limitations remain in Moldova’s current EPR system:

### 1 Aging JEE

The most recent JEE may not fully reflect the current state of Moldova’s preparedness for RN emergencies, as emergency preparedness efforts have been intensified during recent response to COVID-19 pandemic. An updated evaluation is required to reflect the advancements made in the recent past.

### 2 Limited national experience with nuclear emergencies

Moldova’s historical lack of experience with large-scale nuclear programs means that the national health sector has limited experience in managing RN emergencies. This lack of practical experience poses challenges in ensuring that training and preparedness measures are sufficiently robust.

### 3 Gaps in the availability of specialized medical supplies

While progress has been made in establishing national stockpiles, there are still gaps in the availability of specialized medicines for advanced clinical management of patients exposed to radiation. The procurement and management of these critical supplies need to be prioritized to ensure a comprehensive response capability.

## Conclusion

The Republic of Moldova has made significant strides in enhancing its emergency preparedness and response capabilities, particularly in the context of RN emergencies. With the support of WHO, IAEA, and other international partners, the country has begun to develop the necessary infrastructure, training programs, and contingency plans to manage potential radiation hazards effectively.

However, there is still much work to be done. The recent training initiatives, while successful, highlight the need for continuous education, regular drills, and the expansion of national stockpiles of medical countermeasures. Furthermore, the challenges posed by emerging threats, such as the ongoing conflict in Ukraine, underscore the importance of maintaining a state of readiness and improving coordination across sectors.

Moldova’s commitment to strengthening its EPR capabilities, combined with ongoing support from the international community, will be crucial in ensuring the safety and well-being of its population in the face of future RN threats. Continued collaboration, innovation, and capacity-building efforts will be essential to achieving this goal.

## List of Abbreviations

ARS: Acute Radiation Syndrome
BioDoseNet: Biodosimetry Network
CBRN: Chemical, Biological, Radiological, and Nuclear
CRS: Cutaneous Radiation Syndrome
EC: European Commission
EOP: Emergency Operations Plan
EPR: Emergency Preparedness and
Response ETS: EMERGO Train System
EU: European Union
HERCA: Heads of the European Radiological Protection Competent Authorities
IAEA: International Atomic Energy Agency
IACRNE: Inter-Agency Committee for Radiological and Nuclear Emergencies
IHR: International Health Regulations
JEE: Joint External Evaluation
MoH: Ministry of Health
NAPH: National Agency for Public Health
NERIS: Nuclear and Radiological Emergency Response and Recovery
PPE: Personal Protective Equipment
RN: Radiological and Nuclear
SPAR: State Party Self-Assessment Annual Reporting
STAR: Strategic Toolkit for Assessing Risks
UN: United Nations
UNHCR: United Nations High Commissioner for Refugees
WCO: WHO Country Office
WHA: World Health Assembly
WHO: World Health Organization
